# Therapeutic approaches targeting aging and cellular senescence in Huntington's disease

**DOI:** 10.1111/cns.70053

**Published:** 2024-10-20

**Authors:** Asif Ahmad Bhat, Ehssan Moglad, Muhammad Afzal, Riya Thapa, Waleed Hassan Almalki, Imran Kazmi, Sami I. Alzarea, Haider Ali, Kumud Pant, Thakur Gurjeet Singh, Harish Dureja, Sachin Kumar Singh, Kamal Dua, Gaurav Gupta, Vetriselvan Subramaniyan

**Affiliations:** ^1^ Uttaranchal Institute of Pharmaceutical Sciences Uttaranchal University Dehradun India; ^2^ Department of Pharmaceutics, College of Pharmacy Prince Sattam Bin Abdulaziz University Al Kharj Saudi Arabia; ^3^ Department of Pharmaceutical Sciences, Pharmacy Program Batterjee Medical College Jeddah Saudi Arabia; ^4^ Department of Pharmacology, College of Pharmacy Umm Al‐Qura University Makkah Saudi Arabia; ^5^ Department of Biochemistry, Faculty of Science King Abdulaziz University Jeddah Saudi Arabia; ^6^ Department of Pharmacology, College of Pharmacy Jouf University Sakaka Al‐Jouf Saudi Arabia; ^7^ Centre for Global Health Research, Saveetha Medical College, Saveetha Institute of Medical and Technical Sciences Saveetha University Chennai India; ^8^ Department of Pharmacology Kyrgyz State Medical College Bishkek Kyrgyzstan; ^9^ Graphic Era (Deemed to be University), Dehradun, India; ^10^ Chitkara College of Pharmacy Rajpura Punjab India; ^11^ Department of Pharmaceutical Sciences Maharshi Dayanand University Rohtak India; ^12^ School of Pharmaceutical Sciences Lovely Professional University Phagwara Punjab India; ^13^ Faculty of Health, Australian Research Centre in Complementary and Integrative Medicine University of Technology Sydney Ultimo New South Wales Australia; ^14^ Discipline of Pharmacy, Graduate School of Health University of Technology Sydney Sydney New South Wales Australia; ^15^ Centre for Research Impact & Outcome, Chitkara College of Pharmacy Chitkara University Rajpura Punjab India; ^16^ Centre of Medical and Bio‐Allied Health Sciences Research Ajman University Ajman United Arab Emirates; ^17^ Pharmacology Unit, Jeffrey Cheah School of Medicine and Health Sciences Monash University Bandar Sunway Selangor Darul Ehsan Malaysia; ^18^ Department of Medical Sciences School of Medical and Life Sciences Sunway University Bandar Sunway Selangor Darul Ehsan Malaysia

**Keywords:** aging, cellular senescence, Huntington's disease, mutant huntingtin

## Abstract

Huntington's disease (HD) is a devastating neurodegenerative disease that is manifested by a gradual loss of physical, cognitive, and mental abilities. As the disease advances, age has a major impact on the pathogenic signature of mutant huntingtin (mHTT) protein aggregation. This review aims to explore the intricate relationship between aging, mHTT toxicity, and cellular senescence in HD. Scientific data on the interplay between aging, mHTT, and cellular senescence in HD were collected from several academic databases, including PubMed, Google Scholar, Google, and ScienceDirect. The search terms employed were “AGING,” “HUNTINGTON'S DISEASE,” “MUTANT HUNTINGTIN,” and “CELLULAR SENESCENCE.” Additionally, to gather information on the molecular mechanisms and potential therapeutic targets, the search was extended to include relevant terms such as “DNA DAMAGE,” “OXIDATIVE STRESS,” and “AUTOPHAGY.” According to research, aging leads to worsening HD pathophysiology through some processes. As a result of the mHTT accumulation, cellular senescence is promoted, which causes DNA damage, oxidative stress, decreased autophagy, and increased inflammatory responses. Pro‐inflammatory cytokines and other substances are released by senescent cells, which may worsen the neuronal damage and the course of the disease. It has been shown that treatments directed at these pathways reduce some of the HD symptoms and enhance longevity in experimental animals, pointing to a new possibility of treating the condition. Through their amplification of the harmful effects of mHTT, aging and cellular senescence play crucial roles in the development of HD. Comprehending these interplays creates novel opportunities for therapeutic measures targeted at alleviating cellular aging and enhancing HD patients’ quality of life.

## Graphical abstract

Huntington's disease (HD) is a progressive neurodegenerative disorder marked by the gradual decline in physical, cognitive, and mental functions, with aging exacerbating the pathogenic effects of mutant huntingtin (mHTT) protein aggregation. Cellular senescence, driven by mHTT accumulation, contributes to HD pathophysiology through processes like DNA damage, oxidative stress, and inflammation, which worsen neuronal damage. Understanding the interplay between aging, mHTT toxicity, and cellular senescence offers potential therapeutic avenues to mitigate the impact of HD and improve patient outcomes.

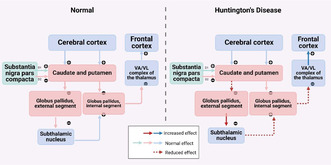

## 1 INTRODUCTION

Huntington's disease (HD) is a progressive neurodegenerative disorder characterized by cognitive deficits, mood swings, and motor impairment.^1^ As an autosomal‐dominant disorder, HD can be caused by a single abnormal gene copy passed from either parent.^2^ The genetic basis of HD lies in the HTT gene on chromosome 4, which encodes the huntingtin protein.^3^ A key feature of HD is the expansion of a cytosine–adenine–guanine (CAG) trinucleotide repeat in the HTT gene.^4^ In healthy individuals, the CAG repeats length ranges between 10 and 35 repetitions.^5^ However, in HD patients, the CAG repeat length exceeds 36, with individuals having 40 repetitions or more being affected by the disease.^6^ This expanded CAG repeat encodes a mutant huntingtin (mHTT) protein that is prone to misfolding and aggregation, ultimately leading to neuronal dysfunction and death.^7^ The occurrence of HD is intricately linked to the processes of CAG amplification, cellular senescence, and neuronal apoptosis.^8^ The expanded CAG repeat in the HTT gene accelerates neuronal aging by inducing cellular stress and damage, which contributes to cellular senescence.^9^ Senescent cells exhibit a permanent cell cycle arrest and secrete pro‐inflammatory factors known as the senescence‐associated secretory phenotype (SASP).^10^ The accumulation of senescent neurons and glial cells in the brain creates a chronic inflammatory environment that exacerbates neuronal damage and promotes neurodegeneration.^11^

DNA damage is both a cause and a consequence of cellular senescence.^12^ The presence of mHTT increases oxidative stress and disrupts mitochondrial function, leading to increased DNA damage in neurons.^13^ This DNA damage activates signaling pathways such as the p53 pathway, which induces cellular senescence and the SASP.^14^ The chronic activation of these pathways further accelerates neuronal aging and contributes to the progression of HD.^15^ Additionally, the inflammatory environment created by senescent cells can enhance DNA damage, creating a vicious cycle that perpetuates cellular senescence and neuronal dysfunction.^16^ Neuronal apoptosis, or programmed cell death, is another critical process in HD pathogenesis.^17^ The accumulation of mHTT protein aggregates disrupts various cellular functions, including transcription, protein homeostasis, and synaptic transmission.^12^ This disruption leads to cellular stress and triggers apoptotic pathways.^18^ The interplay between cellular senescence and apoptosis is complex, as senescent cells can resist apoptosis due to the activation of survival pathways.^19^, ^20^ However, the chronic inflammatory environment and sustained cellular damage eventually overcome these survival mechanisms, leading to neuronal apoptosis and loss.^21^ The relationship between CAG amplification and neuronal aging is further complicated by the observation that DNA damage and oxidative stress can promote the expansion of CAG repeats in the HTT gene.^22^ This somatic expansion of CAG repeats in neurons exacerbates the production of mHTT and accelerates disease progression.^23^ Therefore, neuronal aging not only results from CAG amplification but also contributes to its further expansion, creating a feedback loop that drives HD pathology.

### 1.1 Significance of aging as a critical factor in the progression of Huntington's disease

HD is mostly accelerated by aging because of the cumulative cellular and molecular alterations that intensify the pathogenic consequences of mHTT.^24^ Cellular damage is one of the ways that aging impacts the development of HD as it accumulates with age.^25^ Protein misfolding, mitochondrial malfunction, and DNA damage all increase with age in an individual's cells.^26^ These aging‐related impairments intensify the harmful effects of mHTT in the setting of HD and quicken the degeneration and death of neurons.^27^ Oxidative stress is the critical issue in the pathogenesis of HD. The pathological condition results from an imbalance between the generation of the ROS and the ability of the biological system to either detoxify these chemical intermediates or repair the damage.^28^ Normally, ROS mainly consist of superoxide anion (O2•‐), hydrogen peroxide (H_2_O_2_), and hydroxyl radicals (•OH).^29^ These molecules are reactive and, in turn, can cause damage to the cell components on a great extent leading to neuronal damage and death.^30^ It has been established that mHTT is responsible for the formation of ROS in excessive amounts; therefore, more neurons lose their activity.^31^ Chemically, ROS can oxidize lipids, proteins, and nucleic acids to cause cell damage and activate the cell death pathways.^32^ The lipid peroxidation process can create the reactive aldehydes, malondialdehyde MDA and 4‐hydroxynonenal 4‐HNE, which can react with the proteins and the DNA leading to their impairment, oxidation, fragmentation, and instability.^33^ In addition, ROS can affect another important component of the pathophysiology of HD, that is, mitochondria.^34^ Thus, mHTT can destroy the electron transport chain ETC, reduce ATP production, and increase the generation of ROS.^35^ As a result, such events create a vicious circle of the oxidative process and mitochondrial dysfunction leading to cellular energetic deficiency and neuronal death.^36^ An individual's mHTT further exacerbates the generation of ROS and increases oxidative stress due to its impairment of mitochondrial activity.^37^ A vicious cycle is hereby created by which the oxidative damage tips the balance and eventually quickens the death of neurons and the course of the illness.^38^

Autophagy and proteostasis deficits have a significant influence on the consequences of aging in HD.^39^ These two cellular processes are autophagy and proteostasis which degrade, damaged proteins or organelles cycle with aging get impaired.^40^ However, in HD, along with mHTT, these pathways are disrupted and things start to pile up into dangerous protein aggregates.^41^ With aging, autophagic activity declines, exacerbating the accumulation of mHTT and accelerating neurodegeneration.^42^ Promoting the autophagic flux may be a potential therapeutic strategy to counteract aging in HD patients.^43^ In addition, chronic inflammation, or inflammaging, an aging phenotype also known to affect the development of HD.^44^ Waves of pro‐inflammatory cytokines accompanying aging subsequently exacerbate the inflammatory responses in HD brains driven by mHTT.^45^ This chronic inflammation can undermine the development of HD symptoms as well as neuronal injury. This will enable to discover new therapeutic targets that can alleviate the course of neuroinflammation on aging and HD pathogenesis.^46^

Senescent cells have a characteristic phenotype conveyed to other senescence or non‐senescence cells, referred as the SASP.^47^ As individual cells within a tissue become more senescent, they continue to generate the SASP, which is comprised of many pro‐inflammatory cytokines, chemokines, and growth factors as well as proteases that can markedly change the local microenvironment in tissues.^48^ The rapid accumulation of senescent cells in HD may contribute to exacerbate disease progression, as its associated proinflammatory SASP induces chronic inflammation accelerating neurodegeneration.^49^ A number of such mediators have been detected in the supernatants from p53‐induced senescent cells, for example, interleukin‐6, interferon‐beta, and tumor necrosis factor‐alpha.^50^ They may provoke microglia and astrocytes via positive feedback loop of cytokine stimulation, thus recruiting inflammatory state in central nerves system within the CNS.^51^ The presence of additional inflammatory mediators released by activated microglia and astrocytes results in a feed‐forward loop reinforcing inflammation.^52^ Senescent cells activate matrix metalloproteinases and other proteases which change the extracellular environment.^53^ This remodeling can compromise the structural integrity of the CNS, leading to aberrant migration and propagation of inflammatory signals that promote neuronal dysfunction.^54^ This ECM alteration may hinder the formation and functioning of neural circuits that are crucial for higher brain functions, synaptic function in particular also important for cognitive impairment seen in HD.^55^ Moreover, senescent cells produce promiscuous factors involved in the SASP which are able to recruit peripheral immune cells into CNS, which together with resident microglia aggravate inflammation and tissue devastation.^56^ This recruitment of immune cells may result in a potentiation of inflammation and to the deterioration associated with human HD.^57^

Cellular senescence is an essentially irreversible, permanent state of cell cycle arrest accompanied by deleterious changes in morphology and physiology that involves unwarranted secretion of proteins which promote tissue degeneration and chronic swelling.^58^ In HD, cellular senescence triggered by mHTT induces inflammation in neurons and glial cells that further accelerates neurodegeneration.^59^ In turn, the loss of brain function is further exacerbated by senescent cells that stop tissue from being able to regenerate and repair itself.^60^ A novel approach that could slow the course of HD is to shut down one process: cellular senescence.^61^ Age‐related reduction in neurotrophic support, critically required for survival and function of neurons.^62^ With age, neurotrophic factors such as brain‐derived neurotrophic factor decline.^63^ Lowering BDNF levels contributes to the progression of HD because it renders neurons more vulnerable mHTT toxicity.^64^ Increased neurotrophic support might alleviate the parts of HD and provide greater resiliency to neurons.^65^ Finally, aging‐related genetic and epigenetic changes might lead in some cases to alterations of gene expression patterns and function for genes responsible with stress responses and cellular homeostasis.^66^ It is possible that these alterations synergize with mHTT‐initiated pathogenic processes to promote more rapid degeneration of neuronal health and function.^67^

## 2 PATHOPHYSIOLOGY OF HUNTINGTON'S DISEASE

### 2.1 Huntingtin gene and the cytosine–adenine–guanine repeat expansion

A genetic defect in the huntingtin gene gives daytime for a neurological inherited disease named HD.^68^ The huntingtin protein, produced by the HTT gene on chromosome 4 (4p16.3), is a key molecule for various cellular functions, such as the survival and function of neurons.^69^ The gene is very large, taking up a lot of genomic territory, spans 67 exons, and encodes for a protein that is approximately 3144 amino acids in length.^70^ The expansion of an unstable trinucleotide repeat, CAG, in the first exon of the HTT gene causes HD.^71^ It is essential to understand that this genetic mutation is mostly responsible for the disease.^72^ In normal or non‐carriers, the number of CAG repeats is in the range of 10–35.^73^ The number of these repetitions determines how many glutamine residues (a stretch known as polyQ) is in the huntingtin protein, when the CAG repeat number is over 36, this outcomes in extra‐expanded polyglutamine tract and creates a destructive mHTT protein that causes the advancement of HD.^74^

HD is a fatal genetic disorder that causes the progressive breakdown of nerve cells in the brain.^75^ Patients usually develop signs and symptoms in the prime of their lives, and it mostly affects their motor skills, thoughts, emotions, behavior, and cognition, leading to psychiatric symptoms, dystonia, and voluntary movements.^76^ Across all populations, the prevalence HD is about five to 10 in 100,000 persons, and approximately one in every 10,000 Americans have the disease.^77^ Length of CAG repeat expansion is highly correlated with the pathogenicity of HD.^78^ Whereas nearly all individuals with 40 or more repeats will develop the disease, those who have 36–39 repeats may or may not develop the disease, this is known as reduced penetrance.^79^ When the disease occurs before age 20, it is called juvenile‐onset HD.^80^ This type of the disease is usually associated with a very large CAG expansion, often over 60 repeats.^81^ The enlarged glutamine‐rich tract in mHTT causes a change in the conformation of the protein that promotes its tendency to form aggregates and misfolds.^82^ The intracellular inclusions caused by these aggregates are a characteristic feature of the pathogenesis of HD.^83^ The mutant protein disrupts many cellular functions such as transcription, mitochondria function, axonal transport, synaptic transmission, and others, which is responsible for selective neuronal degeneration mainly in the striatum and in the cortex, and the symptomatology of the disease.^84^

At the molecular level, an extended CAG repeat in the HTT gene triggers a series of harmful effects.^85^ Such mutated protein hinders cellular breakdown services and includes the ubiquitin–proteasome system and autophagy, tampers with regular protein–protein interactions and draws essential transcription aspects.^86^ All these effects trigger neuronal cell death and cellular stress, increased oxidative damage, and the activation of apoptotic pathways.^87^ In addition, the CAG repeats expansion outcomes in genetic instability, particularly throughout DNA duplication and repair, which results in further growth in subsequent generations.^88^ This phenomenon is called anticipation, so affected households have additional earlier onset and more major HD.^89^

### 2.2 Pathogenic mechanisms of mutant huntingtin protein

The HTT gene's enlarged CAG repeat produces the mHTT protein, which is essential to the pathophysiology of HD.^90^ Widespread cellular damage and neurodegeneration are among the adverse outcomes of mHTT.^91^

#### 2.2.1 Protein misfolding and transcriptional dysregulation

The expanded polyglutamine repeat in mHTT predisposes the protein to misfold and aggregate. These aggregates poison neurons by the intra‐cellular inclusions they form.^92^ The accumulation of mHTT causes cellular stress, entrapment of essential proteins and also interference with regular functioning.^93^ Cytoplasmic, neuritic, and nuclear aggregates alter cellular homeostasis and have been involved in neurodegeneration.^94^ mHTT aberrantly interacts with the transcriptional machinery leading to massive transcriptional dysregulation commonly affecting many genes.^95^ It sequesters transcription factors, coactivators, and other regulatory proteins preventing them from performing their regular activities.^96^ These changes affect genes that are critical for the survival and function of neurons, including those involved in energy generation, synaptic transmission, as well as how stress is perceived and transmitted.^97^ Specifically, the transcriptional coactivators CREB‐binding protein (CBP) and other are sequestered by mHTT leading to reduced transcription of neuroprotective genes.^98^

An important manner in which mHTT mediates transcriptional dysregulation is through its binding to a large number of different transcription factors and coactivators.^99^ mHTT interacts with the transcription factor Sp1, thereby leading to changes in expression of a sub‐set of genes controlled by Sp1.^100^ Thus, Sp1 can function in the direct control of synaptic and neuronal genes as well as mediate responses to cellular stress signals.^101^ Sp1‐target gene dysregulation underlies synaptic deficits and stress vulnerability in HD neurons.^102^ One example of an affected gene is BDNF (brain‐derived neurotrophic factor), a vital part in the survival, growth and proliferation of neurons.^103^ The toxic effects of mHTT reduce the expression of BDNF in HD through transcription interference with proteins like CREB (cAMP response element‐binding protein) and CBP.^104^ This lack of BDNF manifests as decreases in neuronal growth and synaptic survival functions, thus amplifying the neurodegenerative state.^105^ The other gene that was revised was PGC‐1α, which is a master regulator of mitochondrial biogenesis and energy metabolism.^106^ Thus, mHTT inhibited the transcriptional activity of PGC‐1α via interference with its binding to other important cellular constituents like transcription factors and coactivators.^107^ mHTT also disrupts the function of REST (repressor element‐1 silencing transcription factor), a modulator that regulates gene expression in neuronal differentiation and synaptic activity.^108^ HD is characterized by a decrease in expression of REST target genes as an effect on synaptic pathology, including BDNF and other synaptophysin (and may be the mechanism underlying restored CREB activity), one Rest‐controlled cellular pathway.^109^ In addition to interacting with ribosomal RNA species, the effects of mHTT on gene expression extend for many genes involved in cellular stress responses as evident by transcriptional dysregulation along entire HSP70 (Heat Shock Protein 70) family.^110^ HSP70s are heat shock protein 70 family chaperones, well‐characterized for their roles in folding newly synthesized proteins and providing cytoprotection under stress conditions.^111^ Thus, lower expression of HSP70 in HD impairs protein folding preventing neurons from properly responding to stress results as higher aggregation and cell loss.^112^

#### 2.2.2 Impaired proteostasis and mitochondrial dysfunction

Mitochondrial dysfunction is a major pathological hallmark of HD and is intricately associated with the neuronal death process.^113^ Mitochondria are multifunctional organelles that primarily synthesize ATP for energy generation, regulate Ca2+ homeostasis, and execute energy‐dependent apoptosis. In cells affected by HD, mHTT proteins impair mitochondrial bioenergetics.^114^ In HD, one prominent feature of mitochondrial dysfunction is the disturbance of the absolute steady state of mitochondrial dynamics, which is maintained throughout the process of fission and fusion in the organelles.^115^ Fission disrupts the mitochondria in a DRP1‐dependent manner, dividing them into smaller sections, whereas fusion, regulated by Mfn1/2 and OPA1, causes strand like, elongated, and interconnected mitochondrial organelles to form.^116^ The study by Song et al., exposed that overexpression of DRP1 in the HD models exacerbated neuronal death. They also reported that the inhibition of DRP1 ameliorated these mitochondrial defects and improved neuronal cell vibrancy. In addition to increased fission, a reduction in fusion is also believed to result in mitochondrial dysfunction in HD.^117^ Reduced fission protein level has been seen in HD Depleted levels of MFN and OPA1, proteins involved in mitochondrial fusion, have also been found in HD, leading to decreased mitochondrial fusion and elongation.^118^ Contrarily expression of Mfn‐2 increased mitochondrial fusion by showing increased elongated mitochondria.^119^ Due to the reduction of fusion and increased fission mitochondrial function and mitogenesis reduced, leading to poor cellular bioenergetics.^120^ Since mHTT inhibits the electron transportation chain which synthesizes 95% of cellular bioenergetics, there is a reduction in ATP and an increase in ROS production.^121^ Moreover, HTT impairs protein denaturation in OCR and the pyruvate kinase step which amplifies energy‐generating deficits precipitating cell death.^122^

**FIGURE 1:**
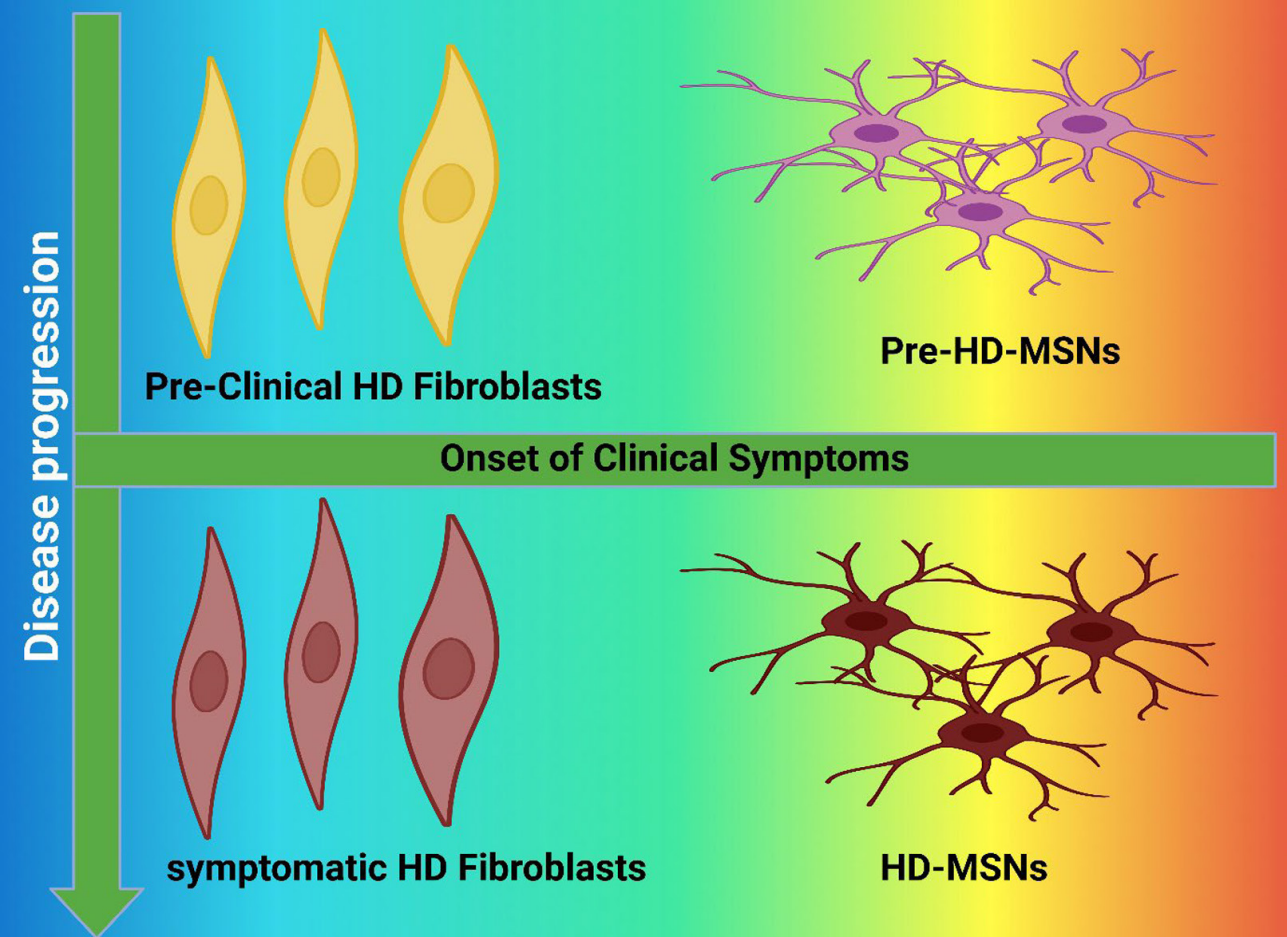
The figure depicts the progression of Huntington's disease (HD), showing pre‐clinical HD fibroblasts and pre‐HD‐MSNs transitioning to symptomatic HD fibroblasts and HD‐MSNs at the onset of clinical symptoms, illustrating cellular changes associated with disease progression.

mHTT can impair not only autophagy but also the ubiquitin–proteasome system, it may influence both components of the cellular proteostasis network,^123^ but there is so much of the mHTT being made that it overwhelms the ubiquitin–proteasome system, which degrades abnormally folded or damaged proteins.^117^ Consequently, these aggregates begin to form, becoming full‐blown hazardous proteosomes.^110^ In addition, mHTT also inhibits autophagy as important autophagic proteins including Beclin‐1 are directly targeted by it.^124^ Autophagy is another important clearance mechanism for cell breakdown, focused on aging and cellular senescence.^125^ Shibata et al., investigated the role of Beclin 1 in controlling mHTT accumulation in HD. They demonstrated a major role of Beclin 1, essential for autophagy and crucial for mHTT clearance. This research is consistent with a model in which mHTT further reduces the autophagic degradation of mHTT by inhibiting Beclin 1‐mediated protein turnover. Moreover, they found that the expression of Beclin 1 reduced with age showing how an age‐dependent decrease in autophagy could favor mHTT accumulation and HD pathogenesis.^126^ Impaired autophagy complicates the build‐up of mHTT aggregates as well as cellular toxicity.^95^ Mitochondrial dysfunction is indeed one of the major features in HD pathophysiology.^127^ mHTT also impairs mitochondrial dynamics, function, and bioenergetics.^128^ Affects both the process of mitochondrial fission and fusion, resulting in aberrant mitochondrial morphology.^129^ It also disrupts the electron transport chain, which decreases ATP synthesis and increases ROS generation by mHTT.^130^ This oxidative stress is yet another reason neuronal death increases, as it literally tears through lipids, proteins, and DNA contained in the cell.^91^ Victor et al. utilized fibroblasts from patients suffering from HD to derive striatal neurons which displayed an age‐related phenotype for studying the effect of aging in HD. The neurons also mirrored an array of HD features that develops over time, including mHTT aggregates within cells, DNA damage, mitochondrial dysfunction, and degeneration. The age signature was altered from fibroblasts or presymptomatic HD cells, reshaping the manifestation of the illness. The results showed the involvement of cellular senescence in the progression of the disease and highlighted how aging is a key factor to consider when modeling HD as shown in Figure 1.^131^

#### 2.2.3 Axonal transport deficits and synaptic dysfunction

Efficient axonal transport of vesicles, proteins, and organelles is crucial for neuronal function.^132^ mHTT disrupts this process by hampering the function of molecular vehicles such as kinesin and dynein.^133^ Cargo‐induced transport deficits result in an accumulation of cargo at the cell body and axon terminals due to defective mHTT‐interaction with these motor proteins, which leads to neuronal dysfunction and degeneration, coupled with multiple blockades in the processes powering synaptic function.^134^ mHTT does affect synaptic plasticity and function, essential to neural transmission as well as cognitive functions.^135^ It impairs synaptic vesicle dynamics, alters the expression of synaptic proteins and disrupts competent neurotransmitter release and recycling.^136^ These changes result in the impairment of synaptic plasticity and transmission which further worsen cognitive and motor impairments characteristic for HD.^137^ Burrus et al. investigated their efficacy in the context of aging and HD. Having deleted mHTT from these SPN subpopulations using the Cre‐Lox system, we also demonstrated that HTT is required for motor control, synaptic formation, and cell viability of SPNs. Loss of mHTT function in SPNs led to substantial degeneration, suggesting that the toxic gain‐of‐function and loss‐of‐function of mutant HTT both play a critical role in the pathogenesis of HD. Our study highlights the importance of HTT in SPN survival along with the effect of aging on HD progression.^138^

Axonal degeneration, which is axonal breakage and loss of connectivity, has long been recognized as a critical component of various neurological diseases.^139^ Wallerian degeneration, in which the distal part degenerates away from the axonal lesion and debris is eliminated by glial cells.^140^ The second type of axonal degeneration called dying‐back degeneration, is common to peripheral neuropathies and starts much distal before retrogradely moving upwards toward the cell body.^141^ This degeneration is a major focus in both research and therapy approaches for neurodegenerative diseases, as it disrupts the connectivity of the brain and therefore plays into how disease pathology manifests as functional impairment.^142^ Li et al., investigated a suitable tool for brain intranuclear early pathogenic events during HD circuits formation. They found that neurophil aggregates primarily occur in the striatal neuron termination site of lateral globus pallidus and substantia nigra was the most preferable region for this phenomenon. As these aggregates were associated with mitochondrial and axonal injury, it suggests that the impairment in axons is an antecedent for HD.^143^

Cellular stress (oxidation, ER toxicity, mitochondrial collapse, and inflammation) in the brain is also related with the neurodegenerative diseases.^144^ Oxidative stress is another leading instance of the role too many reactive oxygen species play in neuronal damage, a key factor seen in both Alzheimer's and Parkinson's disease.^22^ ER stress is induced by misfolded proteins and results in the unfolded protein response.^145^ ER stress is implicated in amyotrophic lateral sclerosis, HD, and Alzheimer's disease.^146^ Parkinson's and HD, which may be due to mitochondrial dysfunction leading to deficiency in energy generation as well raised reactive oxygen species. Long‐lasting neuroinflammation exacerbates cellular injury and is reported to be associated with neurological diseases.^147^ Machiela et al. found that aging increases oxidative stress in mouse neurons, leading to increased DNA damage and heightened susceptibility to mHTT protein. This exacerbates HD pathogenesis, emphasizing the need to address cellular stress and aging to prevent HD progression and counteract cellular senescence.^148^ One common feature among AD, PD, and HD is the accumulation of senescent cells and the associated SASP.^149^ Senescent cells secrete pro‐inflammatory cytokines, chemokines, and proteases, contributing to a chronic inflammatory state in the brain.^150^ This neuroinflammation exacerbates neuronal damage and disease progression in all three disorders. The activation of microglia and astrocytes, leading to sustained inflammation, is a hallmark of aging‐related neurodegenerative diseases.^151^ Aging is also associated with a decline in the cellular mechanisms responsible for protein quality control, including the ubiquitin–proteasome system and autophagy.^152^ This impairment leads to the accumulation of misfolded and aggregated proteins, a characteristic feature of AD (amyloid‐beta and tau), PD (alpha‐synuclein), and HD (mutant huntingtin).^153^ The accumulation of these toxic protein aggregates disrupts cellular function and promotes neurodegeneration. HD is unique among these neurodegenerative diseases in that it is caused by a single genetic mutation—a CAG repeat expansion in the HTT gene encoding the huntingtin protein.^154^ This mutation results in the production of a mHTT protein with an expanded polyglutamine tract.^155^ In contrast, AD and PD are primarily sporadic diseases with complex etiologies involving multiple genetic and environmental factors, although familial forms of AD (mutations in APP, PSEN1, and PSEN2) and PD (mutations in SNCA, LRRK2, and PINK1) do exist.^156^

mHTT also sequesters HAP1 required for microtubule‐based vesicle transport. HAP1 interaction with mHTT itself directly attenuates the trafficking of synaptic vesicles to presynaptic terminals, thus reducing the SV pool for neurotransmitter release.^157^ This disruption of vesicle transport and levels, in turn, underlies the synaptic transmission defects observed in HD.^158^ mHTT impairs the function of proteins in synaptic vesicle fusion machinery, like Soluble N‐ethylmaleimide‐sensitive factor attachment protein receptor (SNARE) proteins.^159^ mHTT interacts with SNARE proteins, syntaxin‐1 and SNAP‐25 where by this interaction it disrupts the assembly of these SNARE complex that facilitates synaptic vesicles fusion toward presynaptic membrane leads to neurons releasing their neurotransmitters. This then results in an impairment of neurotransmitter release, further contributing to synaptic transmission deficits. mHTT also impairs synaptic plasticity, an essential mechanism for learning and memory.^160^, ^161^

Development in synaptic plasticity relies heavily on long‐range potentiation and depression between synapses. mHTT disrupts these processes through interfering with signaling pathways that are required for LTP and LTD induction and consolidation.^162^ It has been reported that mHTT inhibits the function of NMDA (N‐methyl‐D‐aspartate) receptor leading to impaired synaptic plasticity.^163^ mHTT disrupts the migration of NMDA receptor to synapses from a reserve pool and causes alterations in receptor functioning, which impairs synaptic LTP and LTD.^164^ These synaptic alterations in HD more likely than not underlie the majority of its cognitive and motor symptoms as well. In the cortex and hippocampus, synaptic plasticity is impaired but also learning and memory are impacted in a reduction of neurotransmitter release that leads to cognitive deficits.^165^ Chorea and dystonia are motor features seen in early stages of HD, which correlate with basal ganglia synaptic dysfunction since they have a key role on the control of posture movements.^166^ The net effect of impaired synaptic function induced by elevated levels of mHTT contributes to a package deal neural communication deficit capable of explaining anosognosia in HD as well as the slow degeneration that marks its memory and learning impairments.^167^

#### 2.2.4 Cellular senescence

mHTT has been discovered to cause cellular senescence, a state of long‐lasting cell cycle arrest that cells enter in response to various stresses, including oxidative stress, oncogenic activation, and DNA damage.^168^ Cellular senescence is characterized by several distinct features, including changes in chromatin structure, cell shape, and the acquisition of a SASP.^169^ The SASP is a hallmark of senescent cells and involves the secretion of a variety of pro‐inflammatory cytokines, chemokines, growth factors, and proteases.^170^ This secretory profile can induce aseptic inflammation, causing damage to neighboring cells and altering the tissue microenvironment. In HD, the presence of mHTT in neurons and other cell types increases cellular stress and damage, activating signaling pathways such as the p53 pathway that mediate senescence.^171^ This activation leads to the halting of cell division, serving as a barrier to prevent the spread of damaged cells.^172^ While cellular senescence functions as a protective mechanism to prevent the proliferation of damaged cells, its persistent presence can be detrimental, particularly in HD.^173^ Chronic inflammation induced by the SASP can exacerbate neuronal damage and contribute to disease progression.^174^ Molero et al. studied the impact of mHTT on aging and cellular senescence in HD using a mouse model expressing mHTT throughout development. They found that mHTT expression led to striatal neurodegeneration, motor deficits, and abnormal neuronal activity, promoting HD etiology and progression.^175^

Proteolysis, the breakdown of proteins by enzymes, plays a crucial role in maintaining cellular homeostasis by removing damaged and misfolded proteins.^176^ Dysregulated proteolysis leads to the accumulation of such proteins, worsening neurodegenerative diseases. In HD, defects in proteolytic mechanisms result in the accumulation of mHTT, which contributes to neuronal toxicity.^177^ Normal proteolytic activity is essential for the removal of damaged proteins, thereby maintaining cell integrity and preventing the adverse effects of cellular senescence.^178^ Ehrnhoefer et al. found that inhibiting the caspase cleavage site D586 in mHTT hindered autophagy, leading to decreased accumulation of mHTT in aged mice. Their study suggested that enhancing autophagy through dietary interventions, such as intermittent fasting, can reduce cellular senescence and age‐related mHTT toxicity in HD.^179^

### 2.3 Clinical manifestations of Huntington's disease

HD is a progressive neurodegenerative condition, which is characterized by a wide array of clinical manifestations that get worse over the period of time.^180^ The symptoms of this condition can be defined as motor, cognitive, and psychiatric ones. There are several stages, in which the progression of HD is divided, including pre‐symptomatic, early, middle, and late or advanced disease.^181^

#### 2.3.1 Early stages

The term pre‐symptomatic stage refers to the timeframe before any clinical symptoms appear in individuals with a known genetic vulnerability to heart disease.^182^ When examined using neuroimaging and cognitive assessments, minor alterations in brain structure and function may be detected even in the absence of symptoms.^183^ As the disorder advances molecularly and cellularly, this stage may persist for many years.^184^ The early stage of HD, although it may occur sooner or later, often begins between the ages of 30 and 50 years.^185^ Early signs are frequently very mild and may include a mild clumsiness, little involuntary movements, and minor changes in coordination.^186^ In addition, cognitive signs may include difficulties with planning, solving problems, and multitasking.^187^ At this point, one may also notice psychiatric symptoms like depression, irritability, and mild mood swings.^188^

#### 2.3.2 Middle stages

At the intermediate phase of HD, motor symptoms progress. Chorea, which involves jerky, involuntary movements of the trunk, face, and upper and lower limbs with apparent rest between movement, becomes severer and causes a greater degree of disturbance.^189^ Moreover, bradykinesia, dystonia, and impairment of voluntary motor activities can also occur in patients making them more prone to falls and accidents because of issues with walking, speaking, balancing, and so on.^190^ Cognitive decline also speeds up in the middle phase. People may face executive skills such as organization and sequencing of tasks, setbacks with memory and concentration.^191^ Moreover, making decisions becomes more difficult, and patients may exhibit poor judgment.^192^ Inability to perform these cognitive processes and overall cognitive decline can complicate daily life and make it difficult for a person to live unaided. In the middle phase, psychiatric issues also progress.^193^ Patients may have the feeling of becoming socially withdrawn or unresponsive.^194^ The symptoms of anxiety and depression may speed up, and patients become more prone to aggression or irritability.^195^ Mental illnesses such as psychosis and obsessive‐compulsive behaviors may also begin to appear.^196^ In general, these symptoms decrease the quality of life in people making caregiving more complicated.^197^

#### 2.3.3 Late stages

A major motor disorder develops in the last stages of HD.^198^ Chorea may disappear, but bradykinesia, dystonia, and stiffness generally become more pronounced to the point of severely affecting coordinated voluntary movements.^199^ The patients may appear bedridden or be entirely restricted to wheelchairs, requiring full assistance in all their daily needs.^200^ Dysphagia increases dramatically in severity, causing greater risk of aspiration and malnourishment. With cognitive functions deteriorating, the outcome is severe dementia.^201^ Language change, confusion, and lack of memory become more apparent.^202^ Sufferers can no longer recognize familiar faces or do simple cognitive processes, and their caretaker dependence is worse due to the decline in cognitive function.^203^ Psychiatric symptoms still pose a major part of the problem during the last phases.^204^ Patients frequently continue to experience anxiety, depression, and apathy.^205^ They may also become very contentious or violent, delusions and hallucinations are symptoms of psychosis.^105^ Mental and behavioral issues make taking care of the sufferers even more complex.^205^

#### 2.3.4 Terminal stage

The terminal stage of HD is characterized by complete dependence on caregivers.^206^ Patients spend most of their time in a bed‐chair, are unable to speak and usually suffer from severe weight loss and wasting of muscles.^207^ Moreover, while patients do not die from HD itself, they usually develop a range of complications, such as pneumonia, heart disease, furuncle, and other infections. Therefore, measures applied at this stage are mostly palliative.^208^

## 3 ROLE OF AGING IN HUNTINGTON'S DISEASE

### 3.1 Aging influences the onset and severity of HD symptoms

Aging is known to play a substantial role in the initiation and development of Huntington's disease, or HD.^209^ However, its relationship with the latter is complex, given that the bio‐processes tied to aging exasperate the cell and molecular mechanisms that underpin the pathology of HD. It is reasonable to believe that aging affects the level of exposure to the disease as well as the moment of symptom development.^210^

#### 3.1.1 Onset of symptoms

HD is a genetic disorder caused by an expanded CAG repeat in the HTT gene, leading to the production of the mHTT protein.^211^ While individuals are born with the genetic mutation, this syndrome typically appears in individuals in their mid‐adulthood, usually between the ages of 30 and 50.^212^ This ascertained that the disease has an age‐dependent factor and must include age‐related correlation to express the majority of its symptoms.^110^ There is also an inverse correlation between the CAG repeat on gene HTT and the age of symptom onset.^213^ Despite these approximations, the age of HD onset substantially varies even among people levying similar genetic factors.^214^ External factors, including the natural process of age, tend to sway the appearance of the disease; ergo, the aging process as well as other genetic or environmental modalities, affects the manifestation of the disorder.^215^ The more people age, the higher the chances of cellular senescence.^216^ This means permanent exit from the cell cycle and is encompassed by the emergence of SASP.^217^ This includes pro‐inflammatory cytokines, chemokines, and proteases.^218^ HD is validated by mHTT that is known to curtail cellular health and promote senescence and the emergence of the SASP.^219^ Imposing inflammatory circumstances such as an inflamed brain would induce HD beginning when neuronal injury and tissue repair would be disabled.^220^

#### 3.1.2 Severity of symptoms

Aging is associated with a higher level of oxidative stress and a decrease in performance of mitochondria. These age‐related changes serve to worsen the toxic effects of mHTT.^221^ Oxidative stress is a process in which the generation of reactive oxygen species exceeds the ability to detoxify reactive intermediates.^222^ In HD, mHTT also affects mitochondrial function leading to the organelles producing more ROS, which causes even more damage to DNA, other cellular proteins, and lipids.^223^ The overall effect of oxidative stress and decreased productivity of mitochondria rubbing against this increased production of ROS is accelerated neurodegeneration and a worsened progression of HD.^224^ Moreover, aging affects the ability of cells to degrade the amounts of aggregating mutant HTT proteins and damaged cellular organelles.^225^ Proteostasis is the ability of the cell to maintain proper folding, trafficking, and degradation of proteins, autophagy is the processes of removing damaged and dysfunctional cellular structures, such as organelles and protein aggregates, by the cell.^226^ In HD, both are overwhelmed by the production of mHTT in poorly foldable configuration, which means the XRCC1 cells lose the ability to function leading to cell death in larger numbers.^227^ The ability of older people to use proteostasis and autophagy decreases even further, leading to increased progression and severity of HD symptoms in older patients.^228^

Chronic inflammation characterizes the process of inflammaging.^229^ Regarding HD, both the microglia and the astrocytes in the brain participate in the inflammation process that is activated and never turns off, creating the condition of chronic inflammation.^230^ The SASP factors emitted by the senescent cells are also responsible for the inflammatory state.^231^ The pro‐inflammatory processes lead to even more acceleration of the neuronal damage.^232^ The modulation between the M1 and M2 functioning means that the inflammation processes that should affect the CNS in a positive way are inhibited.^233^ Thus, in the inflammation conditions typical to the aging brain, the progression of HD is accelerated, and the symptoms are more severe.^234^ Additionally, aging has a negative impact on the synaptic function and neuroplasticity.^235^ HTT inhibits the synaptic transmission, which is depression between neurons.^236^ It also leads to the decrease of the synaptic proteins' expression that is the background for the synaptic transmission.^237^ The new connections between the neurons of the CNS in the plasticity manner are created due to the proteins.^238^ Aging also slows down the synaptic plasticity, which is the second time a negative impact is put on the function of HD.^239^ As the synaptic utility is reduced, the neurons cannot be compensated by the synaptic synchronizing and, thus, can be the proponent of the disease with slower progression and smaller amounts of dementia.^240^

## 4 INTERPLAY BETWEEN MUTANT HUNTINGTIN, CELLULAR SENESCENCE AND AGING

### 4.1 Intricate relationship between mHTT, cellular senescence, and aging in HD

Mutant huntingtin protein in HD participates in induction of cellular senescence and aging. Its accumulation disrupts a number of cellular processes, such as DNA repair, oxidative stress, and proteostasis.^241^ Such a complex action accelerates the aging’ stop on cell level and results in a phenomenon known as cellular senescence.^242^ Senescent cells not only fail to divide, but also activate secretion of pro‐inflammatory factors.^243^ This myokine secretion causes neuroinflammation and neurodegeneration.^244^ Furthermore, aging leads to accumulation of senescent cells that further amplify tissue dysfunction and degeneration characteristic for HD.^245^ Another factor contributing to neurodegeneration in presence of mHTT is the malfunction of glutamate release.^184^ The mHTT disrupts synaptic function and disturbs vesicular exocytosis of glutamate.^246^ Consequently, glial reuptake of glutamate is impaired, as there is nothing to be taken back. This leads to prolonged stimulation of post‐synaptic neurons and glutamatergic excitotoxicity.^247^ Neurons die as they are overly stimulated by glutamate, the process is facilitated by the fact that NMDA glutamate receptor allows influx of calcium and its subsequent accumulation in mitochondria, leading to cell death.^248^ The glutamatergic excitotoxicity mainly targets striatal medium spiny neurons, further facilitating the motor and cognitive symptoms of HD.^249^ Silva et al. studied the impact of aging on mHTT expression and its influence on cellular senescence. They found that full‐length mHTT expression enhances CaV 2.2 channels, essential for vesicular release, leading to increased glutamate release. This stimulates post‐synaptic neurons for extended periods. However, inadequate glutamate re‐uptake by glial cells results in a gradual accumulation, degrading mHTT's influence over vesicular release and decreasing overall glutamate release, contributing to HD progression and cellular senescence.^250^

Neurons consist of an array of lineages with multiple types of mature neurons and glial cells, with different developmental pathways from neural stem cells to mature cells.^251^ Neural stem cells have the capacity to develop regularly and to expand and differentiate.^252^ These non‐specific cellular cells (NSCs) first form cells of neural progenitor cells NPCs, which finally make neuroblasts, which are predestined to form neurons.^253^ Also, neuroblasts develop into different types of neurons, including excitatory and inhibitory ones.^254^ In addition to that, the NSCs also generate glial progenitor cells, which produce cells like astrocytes, oligodendrocytes, and microglia, essential for neuronal development.^255^ Mehler et al.'s study on aging and developmental components of adult neural populations in mice revealed that early developmental disruption of certain neuron types contributed to HD's pathogenesis and cell aging. The study found age‐dependent decline in motor function, anxiety‐like behaviors, and brain degeneration characteristic of HD, suggesting that early disruption of certain neuron types is crucial for HD's development.^256^ Huntingtin‐associated protein 1 (HAP1) is involved in the HD pathology.^257^ The chaperone promotes the transport of mHTT and its toxicity by interacting with it.^258^ Subsequent to that, HAP1 dysfunction exacerbates mHTT aggregation and cellular toxicity further compounding the disease course.^259^ Additionally, the involvement of HAP1 in intracellular trafficking pathways has linked this protein to the neuronal dysfunction and degeneration that are pathognomonic of HD.^260^ Chen et al. studied differences in HTT and HAP1 expression in mouse and primate brains, focusing on HD. They found that HAP1 and HTT expression are altered in primate brains compared to rodents. However, HAP1 deficiency enhances mHTT‐induced neuron viability in primary primate neurons. HAP1 is crucial for both mHTT toxicity phenotypes, emphasizing the need for care in species‐specific differences in HD pathogenesis and aging‐related cellular senescence.^261^

Soluble mHTT oligomers, or small aggregates of the mHTT protein, are the central components in the pathophysiology of HD.^262^ This is because these oligomers are very noxious to neurons and cause considerable cellular disruption, neuronal malfunction, and neuronal death.^263^ They compromise HD prognosis by impeding protein degradation processes, mitochondrial function, and synaptic transmission.^264^ In addition, as they are able to travel from cell to cell, soluble mHTT oligomers may exacerbate HD condition throughout the brain.^265^ Marcellin et al.'s study explored the role of aging and cellular senescence in the formation and aggregation of mHTT fragments in HD. They found that existing oligomers diminished in young HdhQ150 mice as the age of insoluble aggregate increased. The study suggested that targeting soluble mHTT oligomers could help minimize HD and aging‐associated cellular senescence.^266^ Caspase‐6, a protease operative in apoptosis and cellular homeostasis, is also centrally involved in HD.^267^ However, in HD excessive activation of caspase‐6 causes cleavage of mHTT and generation of toxic fragments.^268^ Ultimately, such fragments can induce neuronal dysfunction and degeneration even further, thereby driving disease pathology.^269^ Caspase‐6‐mediated proteolysis also incapacitates multiple vistas of basic cellular function, such as the synapse and axonal transport, lamenting neuronal failure in HD.^270^ Wong et al. conducted a study on the impact of caspase‐6 (Casp6) absence on HD features in YAC128 mice. They found that body weight, IGF‐1 level, and depression‐like phenotypes could be partially rescued by Ki67‐positive striatal cell protection through Casp6 ablation. Casp6 depletion diminished mHTT‐586 fragments, suggesting that caspase 8 could generate these forms. The study suggested that inhibiting multiple enzymes processing mHTTs can ameliorate aging‐related cellular senescence in HD.^271^

### 4.2 Relationship between mHTT and DNA damage response in Huntington's disease

The mHTT protein in HD is closely associated with DNA damage response (DDR)‐related dysregulation.^272^, ^273^ mHTT can also lead to oxidative stress, by damaging DNA repair mechanisms and allowing for lesions on the DNA.^274^ Furthermore, mHTT disrupts the function of DNA repair proteins and results in changes to chromatin structure which further enhances genomic instability.^275^ In HD, broken DDR can lead to neuronal death and disease aggravation.^276^ Thus, on the one hand DDR can initiate mHTT clearance programs and conversely it is also likely that activation of DDR pathways feed into mHTT pathology.^277^ In HD pathogenesis, RP1 Associated Protein (RPIA5) is involved.^16^ This protein interacts with HTT and influences cellular localization of HTT. In HD, function of RPIA5 is disrupted by the mHTT expression causing defective protein–protein interactions and cellular dysfunctions.^278^ The defect in RPIA5 contributes to HD pathology by perturbing pivotal cellular processes like regulation of gene expression and quality control mechanisms for proteins.^279^ Morozko et al. studied PIAS1's role in HD, focusing on its effects on DNA damage repair and transcriptional activity. They found that PIAS1 is linked to a transcription‐coupled repair complex and serves as a SUMO E3 ligase for PNKP. PIAS1 knockdown corrected transcriptional dysregulation in HD models and rescued mHTT‐impaired PNKP activity. The study suggests that SUMO modification machinery is related to HD DNA repair and transcriptional modulation.^280^

**FIGURE 2:**
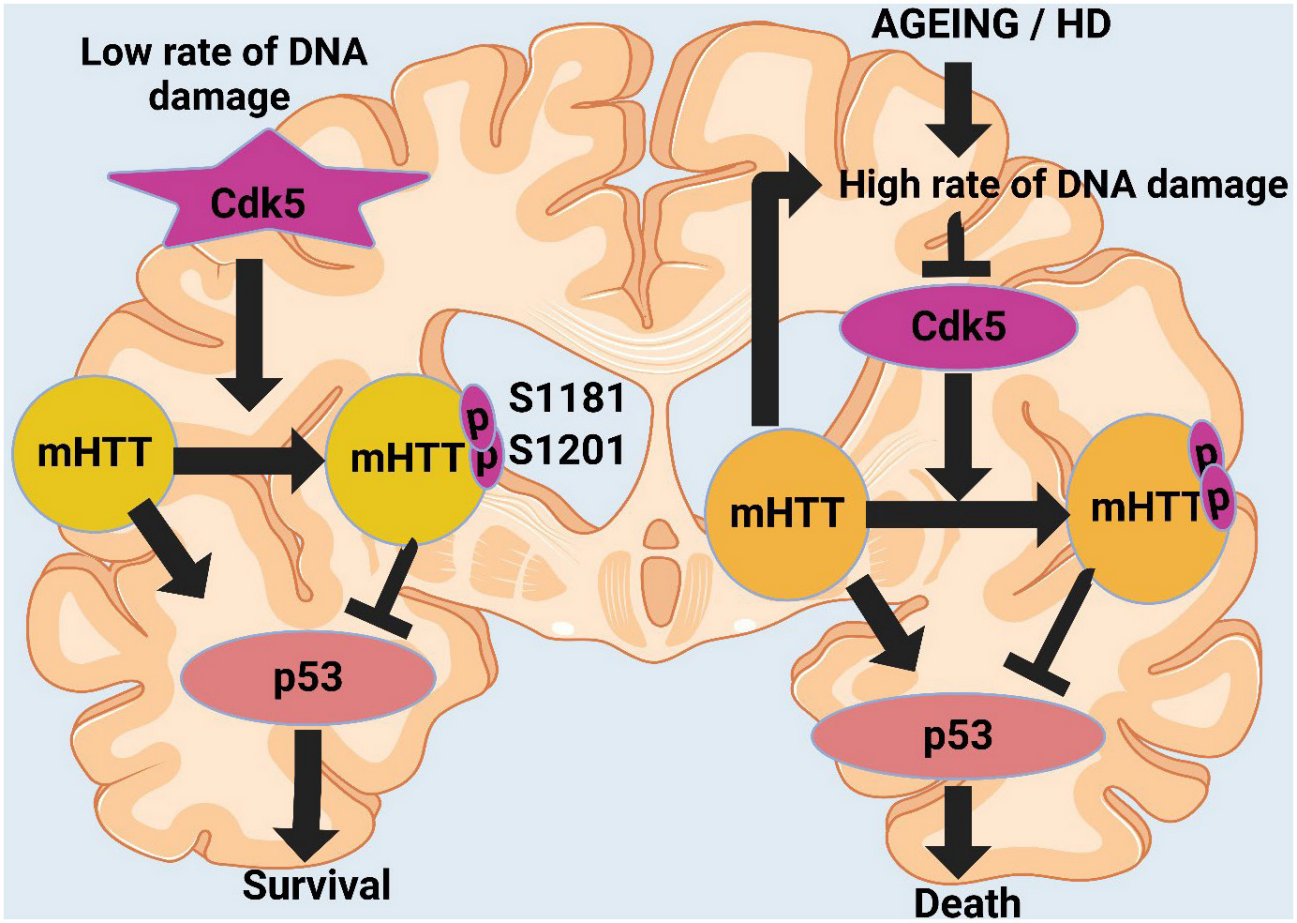
The figure illustrates the differential impact of Cdk5 on mutant huntingtin (mHTT) and p53 under varying DNA damage conditions. In low DNA damage scenarios, Cdk5 phosphorylates mHTT at S1181 and S1201, promoting survival. In aging or Huntington's disease (HD), high DNA damage increases Cdk5 activity, enhancing mHTT toxicity, activating p53, and leading to cell death.

In HD, oxidative damage to mtDNA exacerbates pathology. mHTT impairs mitochondrial localization and function, inducing increased ROS production owing to oxidative damage. ROS‐induced oxidative stress promotes mtDNA mutations and deletions.^281^ This additional mtDNA mutation and deletion compromise leads to more oxidative mtDNA damage and exacerbates HD pathogenesis by enhancing ROS generation and deteriorating neuronal dysfunction and degeneration.^282^ Inadequate mitochondria also cause an imbalance in energy, defective calcium ion metabolism, and altered signaling in cells, heightening stress and contributing to the severity of HD.^283^ Targeting the oxidative damage of mtDNA may be a promising treatment option for reducing mitochondrial dysfunction and reversing apoptosis and autophagy degeneration phenomena in HD.^284^ Siddiqui et al. found that mHTT increased oxidative damage on mtDNA, causing decreased mitochondrial breakdown. This led to increased mtDNA lesions and impaired respiration capacity in HD striatal cells, exacerbated by silencing stress of APE1. The damage is similar in human HD tissues, indicating that oxidative stress and extensive mtDNA affect cellular senescence and neurodegeneration in HD.^285^ HD results in the dysregulation of cyclin‐dependent kinase 5 (CDK5), a critical controller of neuronal development and synaptic activity.^286^, ^287^ In HD, abnormal CDK5 activation can increase neurotoxicity by phosphorylating mHTT, the results of which lead to further toxicity and cause neuronal malfunction and death.^288^, ^289^ Moreover, CDK5 can impair these processes that are fundamental to neurotransmission and cytoskeleton dynamics in the affected cells.^290^ Anne et al. found that huntingtin toxicity is regulated by phosphorylation at serines 1181 and 1201, protecting cells from mHTT‐induced toxicity. Immunohistological analysis revealed sustained DNA damage and downregulation of Cdk5 in late‐stage HD, suggesting dysregulated pathways in DDR and cellular senescence are involved in HD's acute onset to full‐blown form as shown in Figure 2.^291^, ^292^

Neuronal death and neuroinflammation, which can be attributed to the activation of IKK (IκB kinase), are among the mechanisms that underlie HD.^293^, ^294^ Meanwhile, a protein IKK that is usually responsible for controlling inflammation in the body goes haywire in HD, speeding the disease's progress, which then activates IKK in turn, phosphorylates IκB, which means NF‐κB (transcription factor) is released and goes to upregulate pro‐inflammatory genes.^295^ Thus, this chronic neuroinflammation further enhances neuronal injury and aggravates HD pathogenesis.^296^, ^297^ Moreover, IKK activation stimulates apoptotic pathways which in turn lead to the death of neurons.^298^ Khoshnan et al. studied the impact of DNA damage on huntingtin proteolysis, mediated by IKK. They found that DNA damage activates IKKβ to cleave huntingtin, while IKKα counteracts this process. Inhibiting IKKβ and increasing Bcl‐xL reduced caspase activation and huntingtin cleavage, suggesting long‐term neuronal survival in HD.^299^, ^300^

### 4.3 Influence of cellular senescence in glial cells

Maintenance of the healthy and proper function of neurons basically depends on glial cells, astrocytes and microglia in particular.^301^ Senescence induced by expression of mHTT in glial cells has been detected in HD, too.^302^ Senescent glial cells transition to a dysfunctional phenotype that poorly support neurons while secreting high levels of SASPs,^303^ this glial senescence leads to a toxic environment for neurons, which in turn initiates neuroinflammation and contributes to the overall HD pathology.^304^, ^305^ Astrocytes worsen HD symptoms by inducing neuroinflammation by releasing pro‐inflammatory cytokines that form a hostile environment for neurons.^306^ When astrocytes become dysfunctional, they do not support neurons sufficiently and cause synapses to work abnormally and death of brain cells.^307^ Furthermore, astrocytic glutamate transporters break down, which results in excitotoxicity within the neurons that cause more damage.^308^, ^309^ Astrocytes similarly affect the integrity of the blood–brain barrier and as a consequence, injurious substance can penetrate into the brain.^310^, ^311^ These observations suggest that astrocytes play an important part in migration to PC‐responsive living tissues like neuroblasts and leave some remaining queries open about understanding regarding migrants.^312^ The faster death of neurons combined with deficient support and increased neuroinflammation results in more pronounced N855 cell‐death discs recovery accompanied with neurological symptoms, increasing the importance of astrocytes further for advancing HD.^313^, ^314^ Bradford et al. found that mHTT expression in astrocytes worsens neurological symptoms in mice with HD pathology. Astrocytic mHTT‐expressing transgenic mice displayed increased seizure vulnerability to glutamate and worsened neurological disease when crossed with neuronal mHTT‐expressing mice. This suggested that enhancing glial function may be a potential therapeutic strategy for HD.^315^, ^316^

FOXO‐dependent pathway is an extensive contributor to HD development. This process includes various aspects of cells, such as metabolism, cell responses to stress, and death.^317^ At the same time, in HD, an interaction between the mHTT protein and FOXO results in changing the transcriptome profile because of FOXO dysregulation.^318^ The consequence of differences in the transcriptome profile is an essential impact on neuronal cell death as a result of impaired cellular stress responses, increased oxidative stress, and mitochondrial dysfunction.^24^ However, FOXO‐mediated‐transcription is also involved in autophagy, a vital mechanism for removing mHTT aggregates.^319^, ^320^ Martin et al. conducted a genetic screen, revealing that Foxo‐dependent mechanisms protect HD pathogenesis through glia‐specific dCBP depletion. They observed increased locomotion and extended lifespans in HD animals due to decreased glial dCBP levels. This research provides insight into unique pathogenic processes in glia cells, suggesting targeted pathways for efficient HD therapeutics.^321^ In HD, a decrease in glutamate uptake is a significant factor contributing to the pathogenesis of neurodegeneration.^322^ Astrocytes become defective at clearing excess glutamate from synaptic clefts due to the mHTT protein.^323^ The expression and activity of glutamate receptors, specifically GLT‐1, directed at the clearance of extracellular glutamate is reduced.^324^ Consequently, there is an increase in extracellular glutamate leading to excitoxicity and overstimulation of the NMDA receptor in neurons.^325^, ^326^ The overactivity occurs in calcium influx, mitochondrial failure and finally the neurons die. Glial cells in HD are taken to have a significant role in neuronal excitotoxicity.^327^ Shin et al. studied the pathogenesis of HD, arguing that the nuclear accumulation of mHTT protein in glial cells leads to a decrease in glutamate receptor expression. This decrease in glutamate uptake, triggered by mHTT, increases the vulnerability of neuronal cells to excitotoxity. Neurons only died when glial cells started expressing mHTT, suggesting that neuronal cells' susceptibility to excitotoxity was due to decreased glutamate uptake by mutated glial cells.^328^, ^329^

HTT's CAG repeats are directly associated with HD, when they are expanded, the mutant protein gains toxic properties and the ability to form aggregates that disrupt cellular metabolism and, ultimately, result in enormous neuron cell death.^330^ The neurons most affected are the ones in the striatum and cortex.^331^, ^332^ Conforti et al.'s study revealed that NSCs lack huntingtin, allowing them to transform into glial cells. They also found dying neurons with mHTT expression. The study confirmed previous findings, showing that expanded CAG repeats cause extensive cell death and increased caspase activity in NSCs. In HTT‐KO NSCs, GFAP+ glial cells are more prevalent and MAP2+ neurons are lower. mHTT inhibits differentiation into the neuronal lineage.^333^, ^334^

### 4.4 p53 Molecular pathways linking mHTT to senescence

The p53 molecular pathway is the most important mediator of the cellular senescence triggered by mHTT in HD.^335^ mHTT protein disturbs cellular activities by initiating the production of free radicals and subsequently increasing DNA damage.^336^ At high levels of these stressors, the p53 pathway is activated, which is a key pathway that regulates the response of cells to DNA damage and stress.^95^ Therefore, since the production of the ROS is highly activated due to mHTT that affects the mitochondrial activity, p53 is further activated to cleave acetyl groups from histones, therefore playing a stronger role in cellular senescence.^337^ The principle of senescence depends on the increased p53 control of the cell cycle and cellular aging. Therefore, the cell becomes senescent and is recognized as inappropriate.^338^, ^339^ These cells accumulate in the brain and not only enhance its inflammatory condition but also improve degeneration of neurons.^340^ Furthermore, the p53 pathway can interact with other related pathways such as autophagy.^341^ Ehrnhoefer et al.'s research on HD focuses on p53's role in the disease by examining the activation of Caspase 6 in muscle tissue. The study reveals that mHTT upregulates p53, increasing its expression and activation, which facilitates further atrophy in HD by improving muscle cleavage and preparing for apoptosis. Moreover, mHTT lowers the apoptotic threshold to cause cell death by activating p53 in the brain and all neuronal tissues.^342^

Pluripotent stem cells have a perspective role in HD both in research and therapy. These cells can be differentiated into any cell type, including nerve cells, which makes this model effective for studying HD pathogenesis.^343^ Patient‐derived induced pluripotent stem cells [iPSCs] can be used to produce HD‐specific nerve cells, which in their turn can be utilized for studying the disease development and testing various medications.^344^ Nerve cells derived from iPS can also be used for replacing lost neurons thus assisting in restoring lost functions.^345^ Szlachcic et al. developed iPSCs from HD patients to study the role of mHTT. They used RNA interference to silence mHTT, resulting in normalization of p53 levels, which were upregulated due to mHTT. This model has provided insights into mHTT's role in p53 pathway alternations and led to therapeutic approaches targeting HD‐related pathways.^346^ Ubiquitination is one of the main mechanisms through which proteins are assigned for degradation by the proteasome system through attachment of a small protein called ubiquitin. In HD, this process goes rogue and the mHTT protein is abnormally ubiquitinated and forms insoluble aggregates.^347^ These aggregates impair cellular function by sequestering various proteins that are supposed to be ubiquitinated.^348^ When intracellular concentrations of mHTT exceed those of ubiquitin, the degradation of other proteins by a similar mechanism is impaired leading to imbalance in the distribution of proteostasis.^349^ The shortage of proteasomes and the aggregates formed by the mHTT and the sequestered proteins accumulate to toxic proportions further stressing the cellular environment.^350^ Illuzzi et al. investigated the role of mHTT in the p53 pathway by initiating the DDR by exposing cells to toxic proteins. They found that upon exposure to the toxic protein, the cells undergo an increased phosphorylation of p53 at serine 15, altered acetylation at lysine 382 and ubiquitination. All these modifications cause the activation of the DDR leading to the seemingly inevitable progress of HD.^351^

**FIGURE 3:**
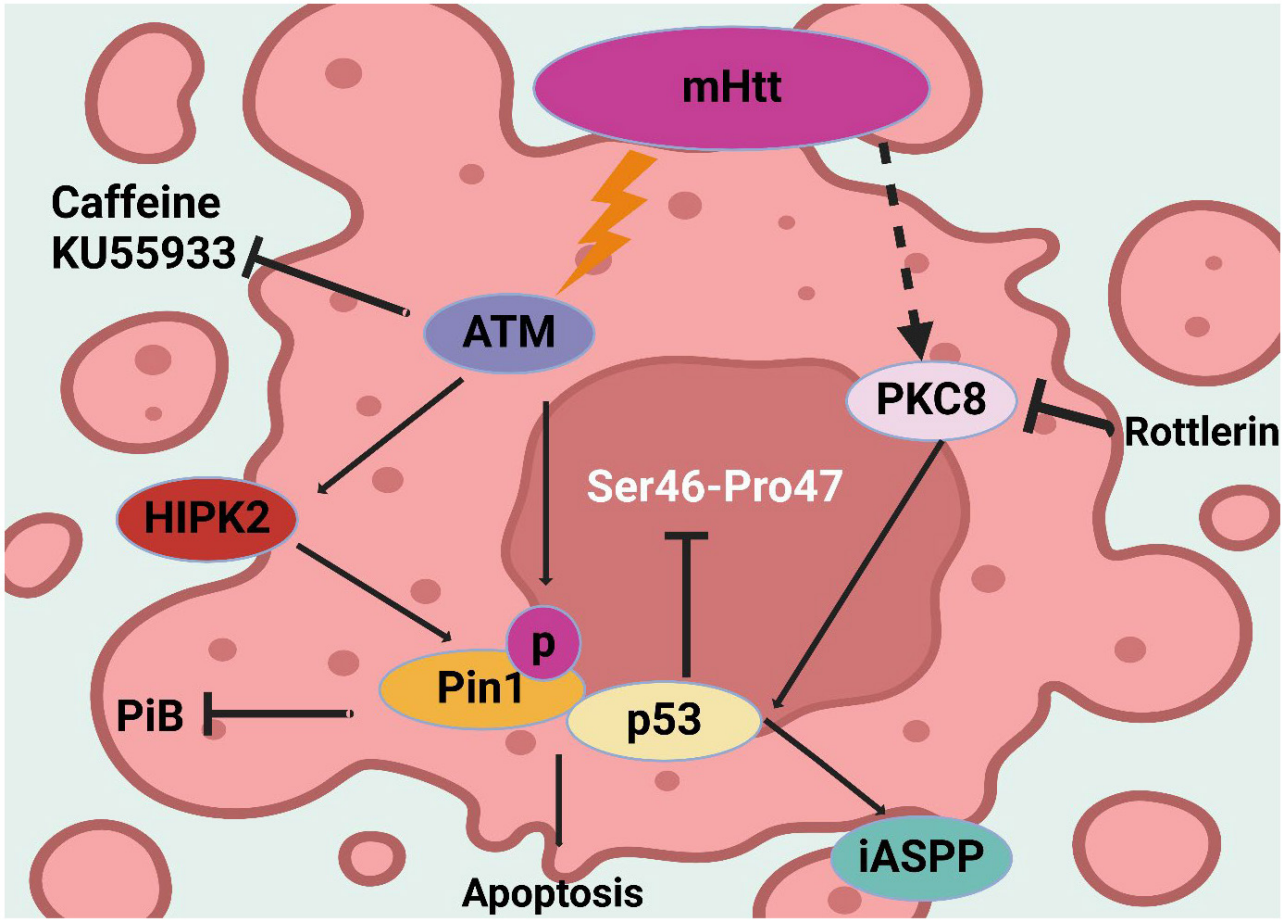
The figure depicts the pathways involving mutant huntingtin (mHTT), ATM, and p53 in inducing apoptosis. mHTT activates ATM, leading to the activation of HIPK2 and Pin1, promoting p53‐mediated apoptosis. PKCδ, also activated by mHTT, facilitates this process. Inhibitors like caffeine, KU55933, PiB, and Rottlerin target these pathways, potentially mitigating apoptosis in Huntington's disease.

One of the most significant phosphorylation sites of the mHTT protein responsible for HD is represented by Ser46.^352^ There is kinase specificity responsible for such type of phosphorylation, and studies show that serine 46 has an impact on the protein toxicity and its ability to aggregate.^353^ When phosphorylated, Ser46 is reported to decrease the ability to form toxic aggregates of the mutant protein and decrease the neuronal toxicity of the protein. In addition, such state is of help to the clearance of mHTT through autophagy.^354^ Grison et al. have conducted their research on the means with the help of which the process of apoptosis is induced with the help of mHTT through the p53 pathway. They determined that mHTT increased the phosphorylation of p53 on Ser46, which made Pin1 bind to p53, and iASPP interact with the p53 protein separately. That, in turn, caused the dissociation of p53 from iASPP, and the subsequent apoptosis. Inhibition of the Ser46 phosphorylation of p53 or the activity of Pin1 prevented the apoptosis as shown in Figure 3; Table 1.^353^

**TABLE 1:** This table summarizes studies on the interplay between mutant huntingtin, cellular senescence, and aging in Huntington's Disease. It includes models used, key findings, mechanisms, cellular changes and therapeutic implications.

Model used	Key findings	Mechanisms	Cellular changes	Therapeutic implications	Reference
HD mouse model	mHTT modulates Cav2.2 channels, age impacts function	Cav2.2 channel modulation	↑ Glutamate release in young	Target Cav2.2 for HD	[355]
HTT ablation in mice	HTT loss causes age‐dependent deficits	Early neuronal disruption	↓ Motor function, brain degeneration	Early intervention in neurons	[256]
Mouse and primate brains	HAP1 exacerbates mHTT neurotoxicity	HAP1 modulates mHTT toxicity	↑ Neurotoxicity in primates	Modulate HAP1 for mHTT toxicity	[261]
HdhQ150 mice	Soluble mHTT forms oligomers, decreases with age	Dynamic mHTT species	↓ Oligomers, ↑ aggregates	Early intervention for mHTT oligomers	[266]
YAC128 mice	Casp6 absence partially rescues HD features	HTT‐586 fragment generation	↓ mHTT‐586 fragments	Target multiple mHTT processing enzymes	[271]
HD models	Polyglutamine expansion alters mHTT conformation	Polyglutamine and phosphorylation	Conformational changes in mHTT	Target mHTT conformational changes	[356]
HD mouse models	mHTT seeding activity increases with progression	Small mHTT structures	↑ mHTT seeding activity	Target small mHTT structures	[357]
Protein interaction network	Rho GTPase signaling modulates mHTT toxicity	Rho GTPase pathway	Altered cell attachment and motility	Target Rho GTPase pathways	[358]
CSF from HD patients	Elevated mHTT seeding species in CSF	mHTT seeding species	↑ Disease onset, neuropathology	Target mHTT seeding species	[359]
HD knock‐in mouse models	Progressive decline in mHTT levels	Differential mHTT levels in brain regions	↓ mHTT in striatum and cortex, stable in cerebellum	Target brain‐specific mHTT levels	[360]
HD models	PIAS1 normalizes mHTT‐affected PNKP activity	PIAS1 modulates DNA repair	Restored PNKP activity	Modulate PIAS1 for DNA repair	[280]
HD striatal cells	mHTT exacerbates oxidative damage to mtDNA	Oxidative stress and mtDNA damage	↑ mtDNA lesions, ↓ respiration	Target oxidative stress	[285]
HD cells	mHTT induces DNA damage response	DNA damage response activation	↑ H2AX, ATM, p53 activation	Target DNA damage response	[361]
Cells expressing mHTT	Cdk5 phosphorylation protects against mHTT toxicity	Cdk5 regulates huntingtin toxicity	Phosphorylation at serines 1181 and 1201	Modulate Cdk5 activity	[291]
IKK‐regulated proteolysis	DNA damage activates IKKβ, leads to huntingtin cleavage	DNA damage influences huntingtin proteolysis	Activation of IKKβ, suppression by IKKα	Modulate DNA damage response	[299]
HD mice	mHTT in astrocytes exacerbates neurological symptoms	Glial mHTT expression	↑ Neurological symptoms	Improve glial function	[315]

Abbreviations: HD, Huntington's disease; mHTT, mutant huntingtin.

## 5 POTENTIAL THERAPEUTIC STRATEGIES TO MITIGATE CELLULAR SENESCENCE IN HUNTINGTON'S DISEASE

Cellular senescence‐mitigating HD presents several plausible therapies to ameliorate senescence‐associated inflammation reduction of cellular resilience and better clearance of senescent cells.^362^ Rapamycin, also recognized as sirolimus, is an immunosuppressant and mTOR inhibitor therapeutically employed in organ transplantation to inhibit organ rejection.^363^ Rapamycin's anti‐proliferative capacity aids cancer therapy by further retarding tumor growth and angiogenesis.^364^ Research has intimated its potential to treat rare genetic disorders such as tuberous sclerosis complex and prolonging life span in relevant animal models suggests it has anti‐aging traits.^365^ Rapamycin acts by binding to a subunit of mTOR, with the protein being fundamental to numerous upstream pathways engaged with cellular growth and metabolic processes.^366^ Roth et al. studied the impacts of rapamycin on aggregation of neuronal mHTT and locomotor performance in a Drosophila HD model. They overexpressed normal and the mHTT proteins in neurons. The results indicated that the mHTT precipitated an aggregation observable with age as well as increased synaptic attrition at the synapses of NMJ in locomotion. The administration of rapamycin mitigated the mHTT aggregation, indicating that the compound could ameliorate the neuronal impairment and peripheral functions.^367^

**FIGURE 4:**
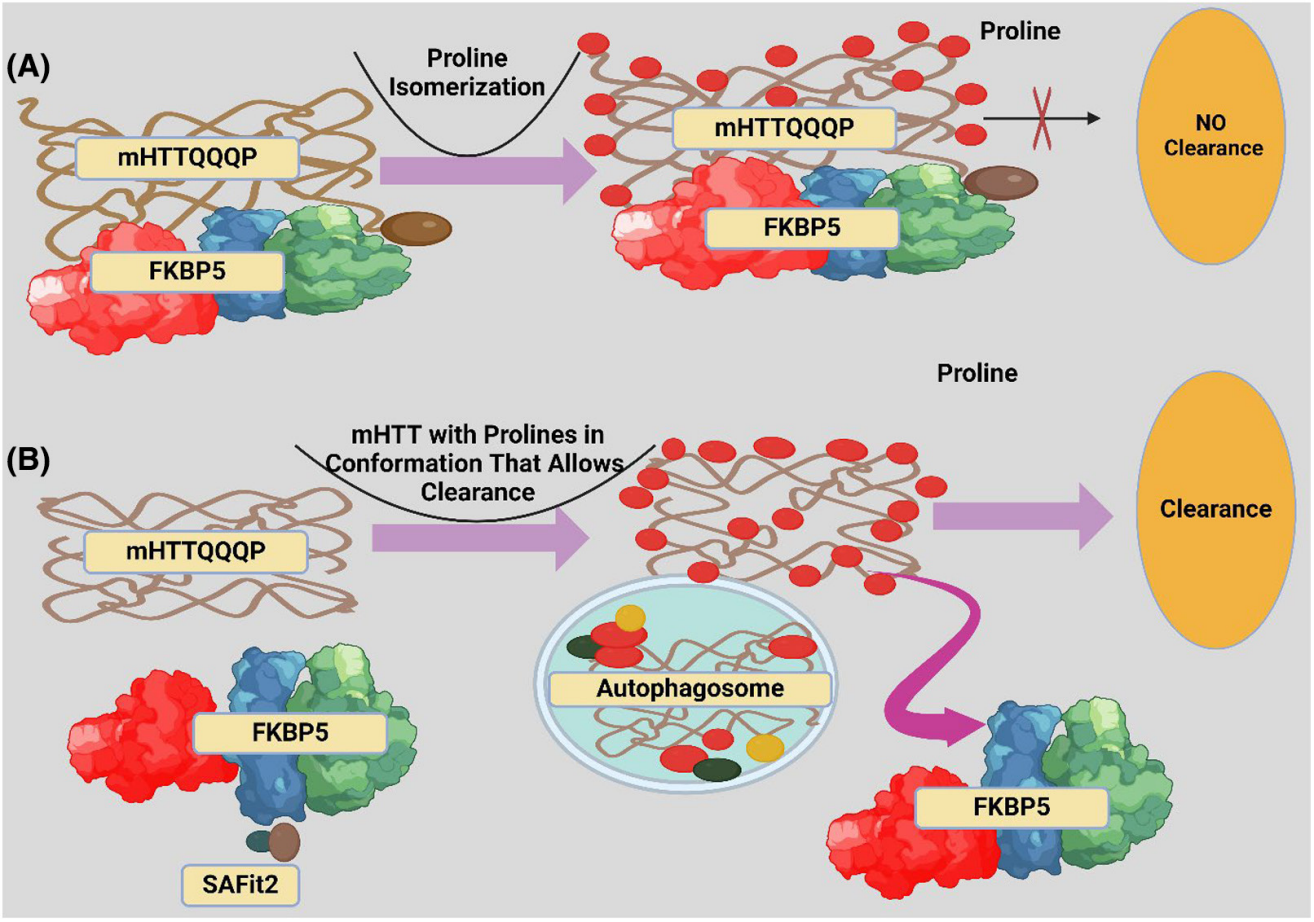
The figure illustrates the role of FKBP5 in the clearance of mutant huntingtin (mHTT) via proline isomerization. In scenario (A), FKBP5 binds mHTT, preventing its clearance. In scenario (B), FKBP5 with SAFit2 facilitates proline isomerization, allowing mHTT to adopt a conformation conducive to autophagic clearance, promoting degradation and reducing toxicity associated with Huntington's disease.

FKBP5 is a protein participating in the regulation of the stress response and has been found to be present at altered levels in HD.^368^ Previous studies have shown that the levels of FKBP5 are elevated in HD patients, sustaining the progression of the disease.^369^ This protein interacts with glucocorticoid receptors, affecting the sensitivity of stress responses and potentially contributing to the destruction of neurons. High FKBP5 levels make such receptors more sensitive, promoting the increased degradation of nerve cells because of stress.^370^ Bailus et al.'s study examined the impact of aging on HD and its effects on FKBP5. They found that FKBP5 levels decrease in aging HD models and human HD cells. FKBP5 interacts with HTT in the brain, and changes in its activity lead to decreased mHTT levels. Improved FKBP5 activity boosts autophagic flux, suggesting a link between mHTT degradation and aging‐associated senescence. SAFit2‐independent FKBP5 inhibition is MTOR‐independent, making trobemovirus FKBP5 a promising therapeutic target for slowing HD progression in aging as shown in Figure 4.^369^ Resveratrol is a polyphenolic compound present in red grapes and a few other plants. It has gained interest in recent years due to its potentially beneficial effects on human health.^371^ Resveratrol is believed to possess antioxidant and anti‐inflammatory properties, which make it potentially beneficial for cardiovascular health, cancer prevention, and longevity.^372^ Using it activates sirtuins, a group of enzymes known to be associated with longevity and the functionality of mitochondria, and resveratrol may mimic the effects of caloric restriction.^373^ High‐quality research such as that by Burger et al. demonstrates that it may enhance insulin sensitivity, prevent or slow down neurodegenerative diseases including Alzheimer's, and reduce inflammation.^374^ A closely related study by Vidoni et al. focused on the effects of this compound on the cells expressing the HD‐causing mutation, using dopamine toxicity as a stressing factor for these cells and focusing on aging and cellular senescence. They showed that resveratrol protects these cells by preserving ATG4‐dependent autophagosome formation and stopping ROS generation and mHTT aggregates processing. These findings can be used to explain the mechanisms behind this compound's beneficial effects and justify its use in therapeutic regimens to slow down HD development and cellular senescence associated with aging.^375^

Green tea is a hot beverage made from the leaves of Camellia sinensis, known for its health effects. It is packed with catechins, a powerful type of antioxidant, especially EGCG.^376^ These benefits include the neutralization of oxidative stress, minimization of inflammation, and improvement in heart health by lowering LDL cholesterol levels.^377^ Regular consumption of green tea can also support weight loss, improved brain function, and a reduced risk of developing cancer, such as breast and prostate cancer as well as some laboratory models of human melanoma, associated with its anti‐carcinogenic properties.^378^ As shown by Varga et al., green tea infusion decreases the severity of HD symptoms by extending the average time to death caused by mHTT protein with prolonged expression of faulty proteins, characteristic of sluggish brain deterioration that results from HD. Thus, overall, these data suggest that consumption of green tea has a small positive effect of being beneficial for preventing and remediating some of the HD symptoms, normalizing some of the signs of aging, and senescence in cells.^379^ Gene therapy is a perspective medical technique that implies genes being replaced or changed in the manner to prevent or treat different diseases.^380^ The approach presupposes the usage of viral vectors or cells editing to get cells equipped with therapeutic genes.^381^ Thus, gene therapy serves for gradual genetic modifications and their elimination or addressing the need to perform specific functions.^382^ This type of therapy can be used for a wide range of conditions and diseases starting from inherited genetic diseases and ending with acquired illnesses like cancer and cardiovascular diseases.^383^ Gene therapies usually touches upon the reasons for a specific disease or predisposition rather than simply treating symptoms meaning that it becomes possible to provide long‐lasting or irreversible results.^384^ Kacher et al. discussed the significance of brain cholesterol metabolism in the HD model. The authors have decided that it would be crucial to pay attention to aging and mHTT to clarify the issue better. The experiments helped to prove that the increase of CYP46A1 allowed for restoring cholesterol homeostasis, improved synaptic activities, and facilitated mHTT clearance. Autophagy became another mechanism facilitated by gene therapy which was proven to serve for neurodegeneration management. The findings and the results of the therapies revealed the benefits of improving the condition of cholesterol metabolism helping to develop new approaches which could reduce cellular senescence associated with aging and neurotoxicity in HD.^144^

Mitochondria‐targeted molecules have been widely discussed as potential solutions in HD owing to strong implication of mitochondrial dysfunction for such diseases.^385^, ^386^ Coenzyme Q10 and MitoQ are types of molecules are targeted to enhance functionality of mitochondria targeting antioxidants, and promotion of enhanced energy production.^387^ The functioning of such molecules cannot reduce oxidative stress promoting oxidative toxicity related to the reduction of toxic activity of the mHTT protein.^388^ This results in damaged syndrome of neurons.^389^ In such a way, the functioning of the targeted molecules can reduce oxidative stress and reduce neuron damages.^390^ At the same time, these molecules can be beneficial at promoting stabilization of mitochondrial membrane and restoration of bioenergetic deficits.^391^ Yin et al. explored actions of mitochondria‐targeted molecules MitoQ and SS31. The authors revealed that mitochondria‐targeted molecules such as MicoO and SS31 rescue mHTT‐induced mitochondrial and synaptic damage. The results of the study were linked to actions of the targeted molecule in aging, and aging related changes in senescence. In addition, such actions were investigated in HD, and the findings of the study demonstrated reduced synaptic damage of striatal neurons expressing mitochondria‐targeted molecules such as MitoQ and SS31.^392^ Manczak et al. similarly examined whether the use of mitochondrial division inhibitor 1 by mHTT else diminished the activity of mitochondria and the process of synaptic functions in neurons. Down‐regulation of fission gene expression used during mitochondrial dynamics was normalized with the use of Mdivi1, and a contradiction was up‐regulated. Moreover, the expression of synaptic gene activity was increased. As these findings reveal, Mdivi1, or other known compound inhibiting the activity of mitochondria and the process of synapse division, protects from mitochondria problems and their death, as well as synaptic degeneration under the attack of mHTT.^393^

Autophagy is a cellular degradation and recycling process that serves to maintain cellular homeostasis by eliminating damaged organelles, as well as misfolded or aggregated proteins.^394^ In HD, the autophagy pathway is impaired causing mHTT protein aggregates to aggregate with subsequent neuronal toxicity and degeneration. A variety of autophagy modulators are explored in preclinical HD models.^395^ These modulators are designed to induce autophagy initiation, promote autophagosome formation or inhibit lysosomal degradation of cargo.^396^ Phosphodiesterase‐3 inhibitor Trequinsin has been recently suggested to increase autophagy and decrease mHTT aggregate formation. A study by Bauer et al. showed that trequinsin ameliorated autophagic flux with a decrease in neurodegeneration in HD mouse models. This compound is a new class of autophagy enhancers that may have therapeutic value for HD.^397^ Lithium, an established mood stabilizer has been reported to increase autophagy by inhibiting inositol monophosphatase (IMPase) and decreasing intracellular levels of inositol. Lithium has been shown to decrease mHTT aggregates in preclinical HD models and improve motor function, as well as provide neuroprotection. A dual role in mood stabilizer and autophagy induction renders lithium an attractive candidate for HD.^398^ Spermidine, a polyamine that occurs naturally in food like sperm‐which‐has been reported to induce autophagy by acetylating important protein targets involved with the autophagic process. Overall, treatment with spermidine resulted in reduced aggregation of mHTT and attenuated neuronal death as well as improved motor performance under different intensifying assays in both HD models. Due to its natural presence and reasonable safety profile, spermidine is an attractive therapeutic option.^399^ Tat Beclin 1 is a peptide that induces autophagy through disruption of the inhibitory interaction between Beclin 1 and Bcl‐2. A recent study suggested that Tat‐Beclin 1 can decrease the formation of mHTT aggregates, attenuate neuronal dysfunction and ameliorates longevity in HD models. This is a targeted‐peptides approach to promote autophagy against HD symptoms^400^ (Table 2).

**TABLE 2:** This table summarizes potential therapeutic strategies to mitigate cellular senescence in Huntington's disease.

Models used	Key findings	Treatment	Cellular changes	Therapeutic implications	Reference
HD models, human cells	FKBP5 modulates mHTT levels	SAFit2	↓ mHTT, ↑ autophagic flux	Targeting FKBP5	[369]
Drosophila model	Rapamycin reduces mHTT aggregation	Rapamycin	↓ mHTT aggregation	Using rapamycin	[367]
Neuronal‐like cells	Resveratrol protects cells	Resveratrol	↓ ROS, ↑ autophagy	Including resveratrol in regimens	[375]
Drosophila model	Green tea reduces neurodegeneration	Green tea	↓ neurodegeneration, ↑ longevity	Green tea consumption	[379]
HD models	CYP46A1 restores cholesterol homeostasis	CYP46A1 gene therapy	↑ cholesterol homeostasis, ↓ mHTT aggregates	Targeting cholesterol metabolism	[144]
HD neurons	MitoQ, SS31 improve mitochondrial function	MitoQ, SS31	↑ mitochondrial function, ↓ synaptic damage	MitoQ, SS31 as agents	[392]
HD neurons	Mdivi1 normalizes mitochondrial dynamics	Mdivi1	↓ mitochondrial fragmentation, ↑ synaptic genes	Using Mdivi1	[393]

Abbreviations: HD, Huntington's disease; mHTT, mutant huntingtin.

## 6 GENE THERAPY FOR HUNTINGTON'S DISEASE

Gene therapy is being developed as a potential method to treat HD because it can address the root cause of this disorder, that is, the mHTT gene.^401^ Advances in gene‐editing technologies, especially CRISPR (Clustered Regularly Interspaced Short Palindromic Repeats)/Cas9‐mediated genome editing have created unprecedented possibilities to target and manipulate the mHTT gene directly; thereby raising hope for disease‐modifying therapeutics.^402^, ^403^ CRISPR/Cas9 is a new and revolutionary gene‐editing tool that provides the ability to produce specific genomic deletions or modifications.^404^ The tool uses a guide RNA (gRNA) to directly the Cas9 nuclease toward specific DNA sequence at which it cripples by induced double‐strand breaks.^405^ The cell's own repair processes patch the break, allowing deletion, insertion or replacement of genetic code. For HD, CRISPR/Cas9 could remove the pathogenic mHTT gene and therefore decrease production of the toxic mutant protein. CRISPR/Cas9 technology has yielded marked success in preclinical models via CRISPR/Cas9 to remove the expanded CAG repeat region of mHTT gene.^406^ It is intended to lessen the toxic effects of the huntingtin protein by lowering the length of its polyglutamine tract.^407^ Approach toward targeting the mHTT gene in allele‐specific manner, since HD patients typically have one normal (wild‐type) and one mutant allele of the HTT gene, it is possible to design CRISPR/Cas9 strategies that selectively target the abnormal allele while preserving expression of wild‐type protein.^408^ Design of gRNAs recognizing single nucleotide polymorphism (SNP) in the mutant allele are designed to be precise, targeting SNP associated with lethal mutation site.^409^

In addition, the improvement of delivery systems has also intensified the possibility for treating HD with CRISPR/Cas9. The problem of efficient, targeted delivery and intracellular uptake of CRISPR/Cas9 components to the affected neurons is still a key one that must be addressed in order for therapeutic efficacy.^410^ For in vivo delivery of CRISPR/Cas9, viral vectors such as adeno‐associated viruses have been extensively utilized. Advancements in AAV engineering have led to enhanced vector specificity, minimizing off‐target events and improving safety.^411^, ^412^ Sun et al. developed neuropathic promoters to deliver CRISPR/Cas9 specifically into the striatal neurons, a vulnerable area in HD which results in effective reduction of mHTT expression and neuroprotection against neuronal death overexpressing pathological mHTT protein toxicity. Furthermore, the research is on progress in finding new delivery methods such as nanoparticles and liposomes to address some of the problems related to viral vectors.^413^ Together with the potential for sustained delivery and reduced immunogenicity, these approaches go a long way toward enabling clinic‐ready CRISPR/Cas9‐based strategies in HD.^414^ The genome editing system based on CRISPR/Cas9 may represent a valuable therapeutic option for HD therapy.^415^ Rapid progress in gene‐editing accuracy, allele‐specific targeting and delivery strategies have started to bear fruit for the development of successful treatment options that address HD's genetic necrosis.^416^

## 7 CHALLENGES, LIMITATIONS AND FUTURE RESEARCH DIRECTIONS

HD is a polygenic and multifactorial neurodegenerative disorder with the expansion of CAG repeats in the HTT gene primarily causing it. This, in turn, results in the production of mHTT.^417^ Unfortunately, the exact cause of the disease is not fully understood, which complicates the creation of effective treatment. Moreover, the role of aging in HD is detrimental, which raises a question of how one's age influences it and which factors can be attributed to HD as a separate matter.^418^, ^419^ A major gap in our understanding is the specific mechanisms by which mHTT leads to cellular senescence. It may be widely accepted that mHTT causes greater cellular dysfunction in HD patients leading to neurodegeneration and all impairments thereof displayed.^420^ Still, there is a significant deficiency in the pathways by, or the manner through which this misfolded protein triggers these responses that are always viewed as hallmarks of senescence, that is, they might or might not occur. In addition, another critical research question might seek further knowledge on the extent that neurodegeneration is associated with these senescence responses.^421^ A more specific branch of this question might be the specific components of the SASP observed in the course of HD that may be the particularly dangerous substances of the various senescence‐associated pro‐inflammatory cytokines, growth factors, and proteases, among others, capable of spurring degeneration, inflammation, or otherwise inhibiting proper tissue repair.^422^, ^423^

Aging is a crucial factor that impacts both the onset and progression of symptoms of HD. Nevertheless, the interplay of age‐associated cellular changes, including reduced mitochondrial function, alterations in proteostasis, and compromised autophagy, and mHTT toxicity is unclear.^424^ In other words, how do changes in the cell's function and structure as influenced by aging affect mHTT aggregation? Such analyses would allow for identifying new targets for intervention that could have an effect on aging and mHTT concurrently.^425^ In particular, while significant progress has been made in the understanding of the effect produced by mHTT on neurons, how nonneuronal cells, including glial cells and other cells of the peripheral system, and other cell types and tissues are affected and contribute to the progression of HD remains poorly understood.^426^, ^427^ In particular, nonneuronal cells are responsible for supporting the health and functioning of neurons, and if they are affected, an individual develops the symptoms. In the context of aging, it is important to understand how mHTT affects the lifespan of nonneuronal cells and the aging of cells in the sympathetic tissue.^428^ New species of biomarkers would be crucial for the early diagnosis of HD. The symptoms of the disease are not expressed in younger patients, and identification of individuals who would significantly benefit from the early initiation of treatment has to use existing biomarkers.^429^, ^430^ However, they are not entirely reliable, and the predictive validity of available biomarkers is moderate to low.^431^, ^432^ In addition, fewer is understood about the distinctions between normal aging and the deterioration of the organism associated with HD for biomarkers that can track HD from the early stages.^433^, ^434^

Some innovative types of imaging, such as advanced MRI, PET scan, and two‐photon microscopy, could offer novel views of the changes in structure and functioning of the brain affected by HD as they develop over time. They can also be used for monitoring disease progression, studying the effects of potentially curative treatments, and for direct, in‐vivo imaging of cells and molecules.^435^, ^436^ Applying such technologies in both preclinical and clinical research can offer unique insights into the interaction between HD and aging.^437^, ^438^ Single‐cell RNA sequencing is used to examine gene expression in individual cells, providing an accurate view of the HD‐affected brain's cellular heterogeneity. scRNA‐seq can be used to identify cell groups targeted by mHTT and to define the molecular hallmarks of senescent cells.^439^ Subsequently, it may be used to assess how these cell types affect disease progression and the efficacy of curative approaches.^440^, ^441^

CRISPR/Cas9 is an effective technology for gene editing with significant potential for treating genetic diseases such as HD. It may be utilized to correct CAG repeat expansion in the HTT gene or modulate other genes within pathways of interest.^401^, ^442^ However, new advances in the field, such as base editing and prime editing, permit an even more precise correction of mutant phenotypes. I would like to see more funding dedicated to the development of CRISPR‐based therapies for HD which would be tested in preclinical studies.^443^, ^444^ Proteomics and metabolomics are highly efficient methods to study the changes in protein and metabolite levels in patients as a result of both HD and aging. These technologies may reveal signaling pathways targeted by mHTT and potential biomarkers that reflect the stage of disease progression.^445^, ^446^ Furthermore, integrating protein and metabolite profiles with other omics data, such as genomics and transcriptomics, may provide a complete picture of the molecular mechanisms involved in HD.^447^, ^448^

## 8 CONCLUSION

In conclusion, the present review has focused on the considerable impact of aging and cellular senescence on the pathophysiology of HD. The analysis of the findings from diverse studies implies that mHTT accumulation aggravates cellular stress, whereas aging further accelerates this process. In particular, the upsurge in mHTT protein levels was associated with increased DNA damage, enhanced oxidative stress, and the interference with autophagy. Due to the aging, these processes proceed at an augmented pace, and cellular senescence is promoted, which deteriorates neurodegeneration. As a result, mHTT not only disturbs crucial cellular functions, but further intensifies the vicious cycle of cellular stress and damage, accelerated by the natural process of aging. Furthermore, mHTT stimulates a senescent phenotype in neurons and glial cells, increasing chronic inflammation and lowering regenerative capacity. Consequently, such damaged and lost neural cells escalate mHTT levels, which expedites the onset and course of HD. The chronic inflammatory milieu is not only detrimental for neurons, but also interferes with brain repair and regeneration. Therefore, neurodegeneration is further accelerated, leading to the marked decline of cognitive and motor function characterizing HD.

Research has also found that mHTT disrupts the critical cellular processes such as mitochondrial function and proteostasis. Mitochondrial function is a key aspect of the cellular metabolism and with mHTT around, the mitochondria's ATP production was decreased while the production of ROS is increased. These harmful cellular changes further aggravated the cellular damage. Impaired proteostasis resulted in the unchecked production of misfolded proteins and defective organelles which are all toxic to the cells, increasing cell death of HD cells. Aging appears to interface these aspects, which while having mHTT present, causes an increase in the deterioration of the cells, and while aging remains unchecked, cellular senescence remains unchecked as well, thus resulting in increased impact of deleterious processes on a non‐resilient cell. As seen, aging, cellular senescence and HD are interconnected and are driving one another forward. This puts into focus the importance of addressing these symbiotic relationships for the development of effective therapeutics. As aging speeds up the toxic effects of mHTT, finding a point of intervention for the acceleration of HD‐like symptoms by mHTT in the host cells provides a plausible route for the designing of therapeutics. This implicates those therapeutics focusing on reducing senescence promoting cues and increasing cellular resilience would possibly create a better quality of life for HD patients. All in all, returning the level of health and vitality in HD patients is a fine line humanity is willing to walk, as any advances in our knowledge and capabilities would result in longer periods of functional independence for HD patients. This insight not only informs the molecular basis for the onset of HD conditions but also provide motivations for the development of therapeutics in the development of potent interventions to tackle the condition in the future.

## AUTHOR CONTRIBUTIONS

Asif Ahmad Bhat, Ehssan Moglad, Muhammad Afzal, Riya Thapa: Concept and design. Waleed Hassan Almalki, Imran Kazmi, Sami I. Alzarea, Haider Ali: Literature search and manuscript editing and review. Kumud Pant, Thakur Gurjeet Singh, Harish Dureja, Sachin Kumar Singh: Conceptual design and writing guidance. Kamal Dua, Gaurav Gupta: Manuscript review. Vetriselvan Subramaniyan: Manuscript edited. All authors approved the final version of the review.

## FUNDING INFORMATION

None.

## CONFLICT OF INTEREST STATEMENT

The authors declare that there are no competing interests.

## Data Availability

Data sharing is not applicable to this article as no new data were created or analyzed in this study.
